# Unidirectional reconnection of an inter-atrial epicardial connection with wide right atrial insertion site: a case report

**DOI:** 10.1093/ehjcr/ytae604

**Published:** 2024-11-21

**Authors:** Hideyuki Hasebe, Yoshitaka Furuyashiki, Kentaro Yoshida, Kazutaka Aonuma

**Affiliations:** Department of Cardiology, Institute of Medicine, University of Tsukuba, Tsukuba 305-8577, Japan; Division of Arrhythmology, Shizuoka Saiseikai General Hospital, 1-1-1 Oshika, Suruga-ku, Shizuoka 422-8527, Japan; Division of Arrhythmology, Shizuoka Saiseikai General Hospital, 1-1-1 Oshika, Suruga-ku, Shizuoka 422-8527, Japan; Department of Cardiology, Institute of Medicine, University of Tsukuba, Tsukuba 305-8577, Japan; Department of Cardiology, Institute of Medicine, University of Tsukuba, Tsukuba 305-8577, Japan

**Keywords:** Ablation, Atrial fibrillation, Case report, Epicardial connection, Unidirectional conduction

## Abstract

**Background:**

The epicardial connections (ECs) via intercaval fibres connecting the right-sided pulmonary veins (PVs) and right atrium (RA) can preclude isolation of the right-sided PVs. Such ECs occasionally have a unidirectional conduction property.

**Case summary:**

A 62-year-old man was referred to our institution for catheter ablation of paroxysmal atrial fibrillation (PAF). Circumferential antral PV isolation was performed via point-by-point radiofrequency (RF) applications. Thirty months after the ablation session, a recurrence of PAF was observed and a second procedure was performed. The right-sided PV was reconnected via an EC. Radiofrequency application at the RA insertion eliminated the EC. Thirty minutes thereafter, the right-sided PVs were reconnected. However, repetitive firings from the right-sided PVs did not conduct to the RA, indicating a unidirectional (RA to PV) reconnection of the EC, which was resolved by RF applications at the PV insertion. This time, the PV insertion of the EC was targeted and the unidirectional reconnection was successfully eliminated. The patient has remained free from any tachyarrhythmias for 3 years.

**Discussion:**

Although the mechanism of the unidirectional conduction property is unclear, source-sink mismatch and anisotropy are likely involved in the mechanism, as with accessory pathways. Electrophysiologists should be aware of the potential for unidirectional reconnection of ECs.

**Learning points**

- Although an optimal ablation strategy for the elimination of epicardial connections (ECs) has not been established, a narrower insertion is likely a preferable target, as with accessory pathways.
- It is sometimes difficult to notice the unidirectional conduction property of an EC. Electrophysiologists should be aware that ECs can reconnect unidirectionally.

## Introduction

Pulmonary vein (PV) isolation is widely accepted as the cornerstone of catheter ablation for atrial fibrillation (AF). Previous anatomical and electrophysiological studies reported that the existence of epicardial connections (ECs) involving the PVs can preclude PV isolation.^1–4^ A study by Barrio-Lopez *et al*. reported that the prevalence of the ECs involving the PVs was 13.5%. Among the ECs, the prevalence of those involving the right-side PVs was reported to be 7%^3^ and 15%.^4^ Although the electrophysiological characteristics of the ECs have not been fully elucidated, previous studies reported that parts of the ECs have a unidirectional conduction property.^5–7^ In the present case, an EC between the right PVs and RA with a wide RA insertion site was successfully eliminated by radiofrequency (RF) applications at the RA insertion site. However, the EC reconnected unidirectionally, which was resolved by RF applications at the PV insertion site.

## Summary figure

**Figure:**
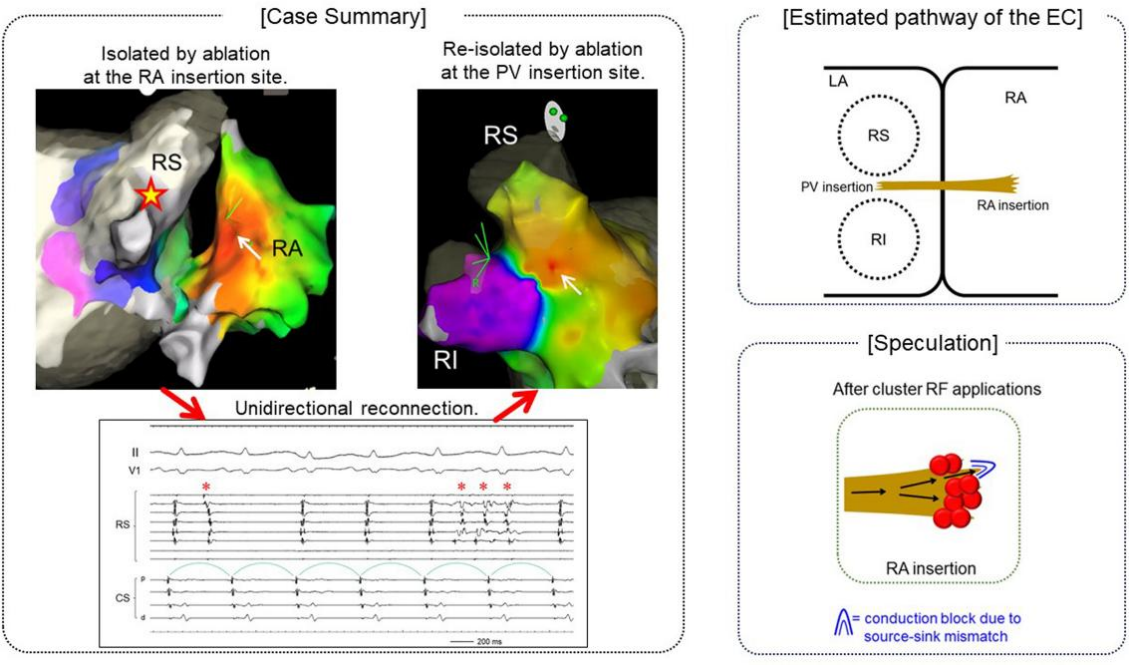


## Case report

**Figure 1:**
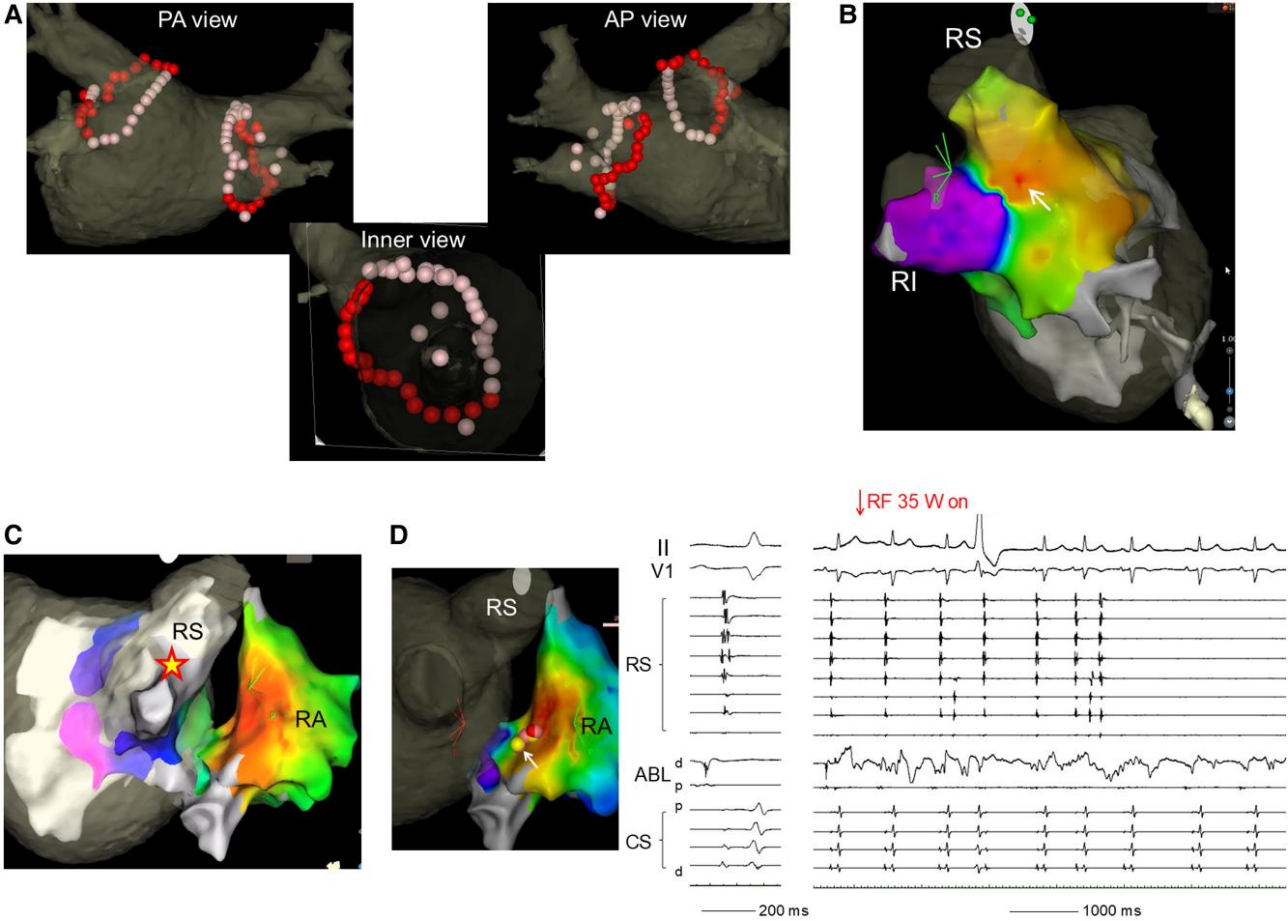
*(A)* Radiofrequency lesion sets in the index procedure. As for the right-sided pulmonary vein, radiofrequency energy was delivered at a power setting of 45 W and a force time integral of ≥600 g·s at the anterior antrum (dense tags) and using catheter dragging technique at the posterior antrum (light tags). *(B)* Activation map in the left atrium during sinus rhythm before ablation. The white arrow indicates the carina breakthrough. *(C)* Activation map of both atria during pacing from the right-sided pulmonary vein carina. The earliest site was the posterior right atrium. The yellow star indicates the pacing site. *(D)* Three-dimensional image and intracardiac electrograms at the successful isolation of right-sided pulmonary vein by radiofrequency applications from the posterior right atrium. High-frequency potentials were recorded by the ablation catheter, and the fourth radiofrequency application (arrow) successfully isolated the right-sided pulmonary veins. ABL, ablation catheter; CS, coronary sinus; d, distal; p, proximal; RI, right inferior pulmonary vein; RS, right superior pulmonary vein.

**Figure 2:**
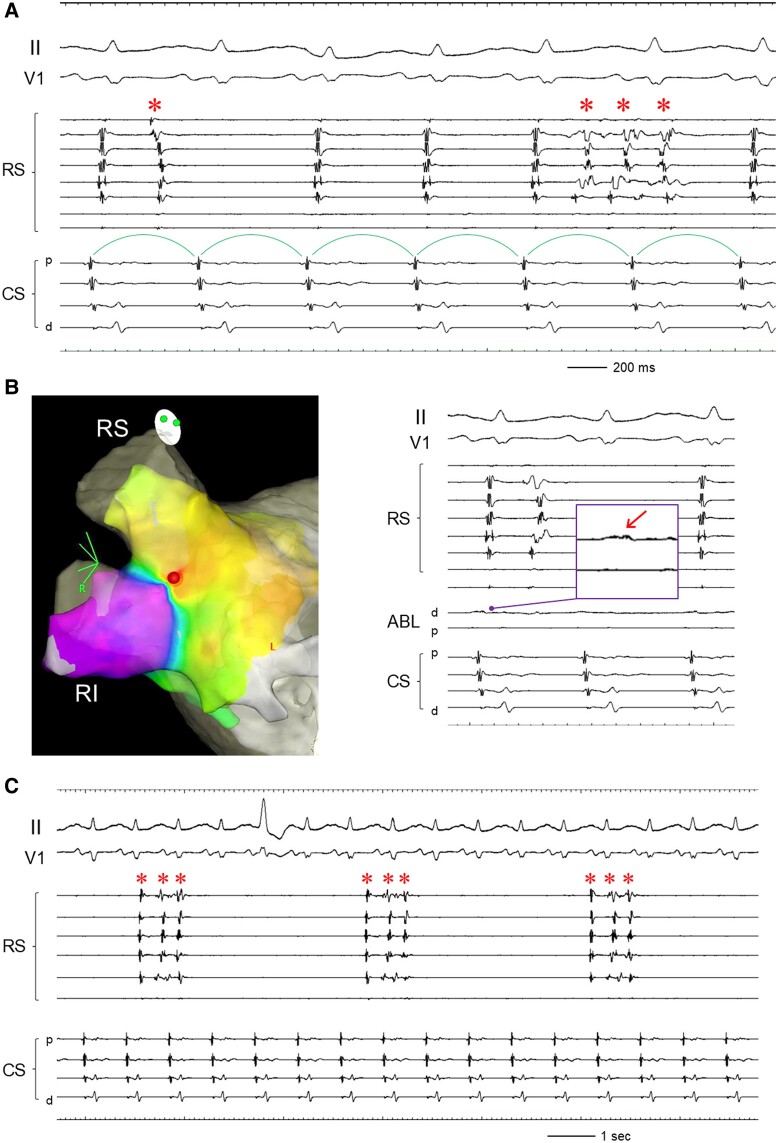
*(A)* Intracardiac electrograms at the reconnection of the right-sided pulmonary vein. Repetitive firings (asterisks) from the pulmonary vein did not conduct to the atrium, indicating a unidirectional reconnection of the right-sided pulmonary vein. *(B)* Three-dimensional image and intracardiac electrograms at the radiofrequency application site (tag) at the PV carina. The local electrogram at the radiofrequency application site is enlarged. *(C)* After the re-isolation of the right-sided pulmonary vein, the repetitive firings (asterisks) inside the pulmonary vein were still observed. Abbreviations are as in *Figure 1*.

A 62-year-old man was referred to our institution for catheter ablation of symptomatic paroxysmal AF (PAF). The patient had hypertension and mild left ventricular hypertrophy (wall thickness was 12 mm in echocardiography). On chest auscultation, there were no notable abnormalities in heart sounds or breath sounds. The ablation procedure was performed using a three-dimensional navigation system (CARTO 3, Biosense Webster, Inc., Diamond Bar, CA). Circumferential antral PV isolation (CAPVI) was performed via point-by-point RF applications with a ThermoCool SmartTouch irrigated-tip contact force-sensing ablation catheter (Biosense Webster, Inc.). After the left-sided PVs were successfully isolated, CAPVI for the right-sided PVs was performed at a power setting of 45 W and a force time integral of ≥600 g·s at the anterior antrum and using catheter dragging technique at the posterior antrum. Even after the encircling RF applications, the right-sided PVs were still not isolated, and additional RF applications of 15–20 s at a power of 35 W inside the antral line were needed (*Figure 1A*). Thirty months after the ablation session, a recurrence of PAF was observed and a second procedure was performed. The right-sided PVs had reconnected, and the activation map during sinus rhythm revealed a breakthrough at the PV carina (*Figure 1B*). An activation map obtained during pacing at a power of 5 mA at 2.0 ms from the mid-carina of the right-sided PVs with a Lasso catheter (Biosense Webster, Inc.) revealed that the earliest activation site was the posterior RA and the activation conducted to the left atrium through the Bachmann’s bundle, indicating that the right-sided PVs had reconnected via an EC and a gap along the antral encircling line was denied (*Figure 1C*). Radiofrequency applications at a power setting of 35 W were delivered at the earliest site in the posterior RA (RA insertion of the EC). The fourth RF application resulted in isolation of the right-sided PV (*Figure 1D*). Additional RF applications were delivered around the successful site to prevent reconnection. Thirty minutes thereafter, the Lasso catheter was repositioned into the right superior PV (RSPV) and showed a reconnection of the right-sided PVs. On the other hand, repetitive firings from the RSPV did not conduct to the atrium. Neither pacing from the PV carina did not conduct to the atrium, indicating a unidirectional reconnection of the EC (*Figure 2A*). This time, we carefully referred the activation map during sinus rhythm and targeted the breakthrough site during sinus rhythm at the right-sided PV carina (PV insertion of the EC). Radiofrequency application of 20 s at a power of 35 W at this site eliminated the unidirectional reconnection of the EC (*Figure 2B*). Additional two RF applications with similar energy were delivered on both sides of the successful site. After the re-isolation, the repetitive firings inside the RSPV were still observed (*Figure 2C*). No further reconnection was observed even with isoproterenol infusion. The patient has remained free from any tachyarrhythmias for 3 years.

## Discussion

Several previous studies reported the presence of muscular connections between the right-sided PVs and RA (i.e. ECs). The insertions of the ECs may vary anatomically and electrophysiologically depending on the case, as with accessory pathways. In our patient, we targeted the RA insertion first considering the risk of PV stenosis due to RF applications at the PV carina. The EC reconnected despite the cluster RF lesions created at the posterior RA. The activation map during sinus rhythm showed a distinct narrow range of activation at the earliest site in the right-sided PV carina, indicating that the PV insertion of the EC was a narrow band. In contrast, the earliest site in the posterior RA on the activation map during pacing from the carina of the right-sided PV was obscure and wide ranging, implying that the RA insertion was a wide band. Although an optimal ablation strategy for the elimination of ECs has not been established, a narrower insertion is likely a preferable target, as with accessory pathways. In hindsight, it may have been better to ablate the PV insertion first rather than the RA insertion in our patient.

A reconnection only in the RA to PV direction may not necessarily need to be eliminated. However, we delivered additional RF applications to eliminate the EC, considering the possibility of its development into a bidirectional reconnection. At the time, we targeted the PV insertion because the RA insertion was considered to be oedematous due to the previous delivery of cluster RF applications.

**Figure 3:**
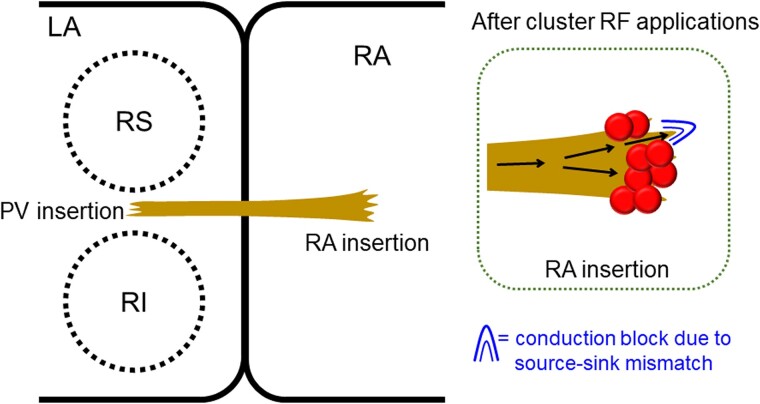
Potential schema of the intercaval bundle. Tags represent radiofrequency applications at the posterior right atrium. Abbreviations are as in *Figure 1*.

Although the mechanism of the unidirectional conduction property is unclear, source-sink mismatch and anisotropy are likely involved in the mechanism, as with accessory pathways.^7^ Although elimination of an EC by RF applications at the RA insertion is often achieved, it is not difficult at the PV insertion.^3,4^ Therefore, an RA insertion with a wide band and a PV insertion with a narrow band might be common characteristics of an EC. A conduction block due to source-sink mismatch is more likely to occur at a boundary where a wavefront propagates from a small volume source to a large volume sink.^8^ Yoshida *et al*.^7^ reported that unidirectional conduction was from the PV to RA direction in all three patients with an intercaval EC with unidirectional conduction property, suggesting that from the RA to PV direction, unidirectional conduction block occurs at the PV insertion at which a fine fibre connects to the large mass of the PV muscular sleeve. In the first place, unidirectional conduction from the atrium to the PV is rare not only to ECs.^9^ In this regard, it is curious that the unidirectional conduction of the EC was from the RA to PV direction in our patient. The EC was reconnected after the cluster RF applications at the RA insertion, suggesting that the unidirectional reconnection occurred within a limited part (i.e. within a narrow range) of the RA insertion. In such a condition, a source-sink mismatch at the RA insertion could have caused a conduction block from the PV to RA (*Figure 3*).

At the time of the reconnection, we might not have noticed the exit block from the PV to RA (i.e. unidirectional conduction) without repetitive firings from the PV. If the unidirectional reconnection had been in the opposite direction (PV to RA), it would have been difficult to recognize the reconnection itself because there had been no PV potentials other than PV firings. The firings from the right-sided PVs would have resembled non-PV triggers from the RA.^5^ Electrophysiologists should be aware of the possibility that ECs can be unidirectionally reconnected.

## Conclusions

The three-dimensional maps in the present patient suggested that the RA insertion of the EC was a wide band, whereas the PV insertion was a narrow band. Although cluster RF applications initially eliminated the EC, it reconnected unidirectionally. Thus, it might have been better to ablate the narrower insertion of the EC first. Further investigation is required to determine the best ablation strategy for the elimination of ECs. Electrophysiologists should be aware of the potential for unidirectional reconnection of ECs.

## Data Availability

The data underlying this article will be shared upon reasonable request to the corresponding author.

## Lead author biography

Hideyuki Hasebe, male, clinical electrophysiologist, graduated from the Faculty of Medicine, Yamanashi University, in 2002. From 2002 to 2003, he worked at Yamanashi Medical University as a resident. From 2003 to 2005, he worked at Shizuoka Red Cross Hospital as a resident. From 2005 to 2006, he worked in the Division of Cardiology at Fujieda Municipal Hospital as a fellow. From 2006 to 2008, he worked in the Department of 2nd Internal Medicine at Yamanashi Medical University Hospital as a staff. From 2008 to present, he has worked in the Division of Arrhythmology at Shizuoka Saiseikei General Hospital. In 2014, he became a director of division of arrhythmology.
